# Putting the N in dinoflagellates

**DOI:** 10.3389/fmicb.2013.00369

**Published:** 2013-12-04

**Authors:** Steve Dagenais-Bellefeuille, David Morse

**Affiliations:** Département de Sciences Biologiques, Institut de Recherche en Biologie Végétale, Université de MontréalMontréal QC, Canada

**Keywords:** dinoflagellates, diatoms, nitrogen metabolism, nitrogen stress, autotrophy, mixotrophy, heterotrophy

## Abstract

The cosmopolitan presence of dinoflagellates in aquatic habitats is now believed to be a direct consequence of the different trophic modes they have developed through evolution. While heterotrophs ingest food and photoautotrophs photosynthesize, mixotrophic species are able to use both strategies to harvest energy and nutrients. These different trophic modes are of particular importance when nitrogen nutrition is considered. Nitrogen is required for the synthesis of amino acids, nucleic acids, chlorophylls, and toxins, and thus changes in the concentrations of various nitrogenous compounds can strongly affect both primary and secondary metabolism. For example, high nitrogen concentration is correlated with rampant cell division resulting in the formation of the algal blooms commonly called red tides. Conversely, nitrogen starvation results in cell cycle arrest and induces a series of physiological, behavioral and transcriptomic modifications to ensure survival. This review will combine physiological, biochemical, and transcriptomic data to assess the mechanism and impact of nitrogen metabolism in dinoflagellates and to compare the dinoflagellate responses with those of diatoms.

## INTRODUCTION

Dinoflagellates are unicellular eukaryotes that appeared ~400 MYA and still thrive today in most marine and freshwater ecosystems (Fensome et al., 1999). They have evolved various life styles, which has enabled them to populate a great diversity of ecological niches. Many dinoflagellates are found within the phytoplankton, and are important contributors to oceanic primary production. Others, such as *Pfiesteria* or *Protoperidinium*, are predators that are known to feed on a wide array of prey. Still other dinoflagellates can be symbiotic, as exemplified by the endosymbiotic associations formed between *Symbiodinium* and some anthozoans. This mutualistic symbiosis is of immense ecological importance because many tropical reef corals live in nutrient-poor water and the photosynthetic products supplied by the zooxanthellae symbionts are essential for growth and survival of the host (Davy et al., 2012). The order Syndiniales is comprised exclusively of parasitic species that infect tintinnid ciliates, crustaceans, dinoflagellates and fish (Park et al., 2002; Skovgaard et al., 2005; Gestal et al., 2006; Harada et al., 2007; Guillou et al., 2008; Small et al., 2012). Curiously, some dinoflagellate genera, such as *Gambierdiscus*, *Ostreopsis* or *Prorocentrum*, can live fixed to a substrate. They can be found both in epiphytic associations with macroalgae and in benthic sediments (Vila et al., 2001). The benthic zone also contains dinoflagellate temporary or resting cysts. It is now believed that one explanation for the ecological versatility of dinoflagellates comes from the three trophic modes, autotrophy, mixotrophy, and heterotrophy they have evolved to harvest energy.

Traditionally, dinoflagellates have been categorized as either photoautotrophic or heterotrophic, based on the presence or absence of chloroplasts. Over the past 30 years, however, it became evident that these two trophic modes were actually the extremes of a continuum, with the middle region being composed of mixotrophic species. Mixotrophs combine photosynthesis and food ingestion to harvest both energy and nutrients, and are quite common in marine phytoplankton, with the diatoms being a noteworthy exception (Stoecker et al., 2009). Mixotrophy can be found in all dinoflagellate orders, even if evidence is stronger in some taxa (Stoecker, 1999). Most dinoflagellates have complex life cycles, and in some cases mixotrophic behavior is only apparent in some life stages. For example, *P. piscicida* lacks chloroplasts and is heterotrophic for most of its life, except in its flagellated zoospore stage where the cells contain functional kleptochloroplasts stolen from ingested cryptophytes (Steidinger et al., 1996; Stoecker, 1999).

Although dinoflagellate life styles are diverse, all species require carbon (C), phosphorus (P) and nitrogen (N). Of these, N nutrition is of particular interest, because high concentrations of various N sources are often correlated with the appearance of harmful algal blooms (HABs) dominated by dinoflagellates (Lee, 2006; Anderson et al., 2008, 2012b; San Diego-McGlone et al., 2008; Zhou et al., 2008). There is a general scientific consensus that HAB events have globally increased in frequency, magnitude and geographic extent over the last 40 years (Anderson et al., 2012b). Concurrently, the impacts of HABs on public health, tourism, fisheries and ecosystems have also increased. Some HABs are toxic, such as those caused by the widespread *Alexandrium* genera, as they can synthesize a suite of paralytic shellfish toxins (PST; Wang, 2008; Anderson et al., 2012a). These toxins accumulating within filter-feeding mollusks can cause paralytic shellfish poisoning. PSTs all contain N and their concentration within *Alexandrium* cells can increase up to 76% following N-enrichment (Hu et al., 2006; Wang, 2008). A better understanding of N metabolism in dinoflagellates could help to better predict the occurrence of HABs and limit their impact.

**FIGURE 1:**
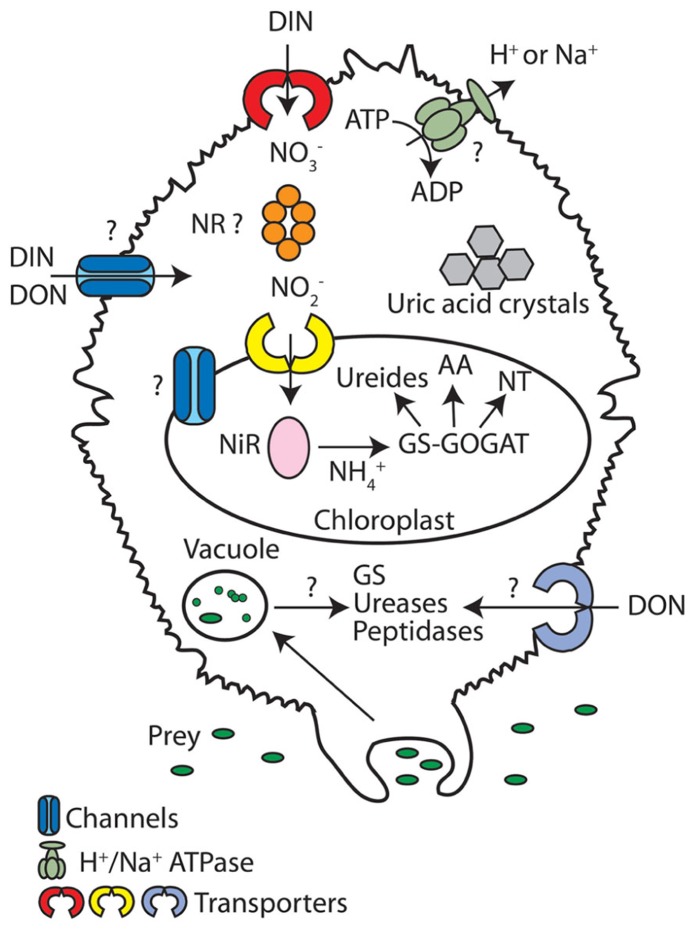
**Nitrogen metabolism in dinoflagellates.** In this schematic overview of N metabolism, transporters and channels are depicted as individual proteins that transport a range of N forms, even though transporters/channels are normally selective for particular N forms. A (?) signifies that the pathways or proteins have not been reported in dinoflagellates, but can be present in other photosynthetic eukaryotes. The (NR ?) indicates that while this enzyme is usually localized to the cytoplasm, a study in *L. polyedrum* indicated it was found to the chloroplast (Fritz et al., 1996). AA, amino acids; DIN, dissolved inorganic nitrogen; DON, dissolved organic nitrogen; GS, glutamine synthetases; GOGAT, glutamine 2-oxoglutarate amidotransferase; ${NH}_{4}^{+}$, ammonium; ${NO}_{3}^{-}$, nitrate; ${NO}_{2}^{-}$; nitrite; NiR; nitrite reductase; NR, nitrate reductase; NT, nucleotides.

This review will cover N metabolism in dinoflagellates of various marine life styles. Unfortunately, even though some physiological and transcriptional studies are available, there is little known about the molecular components involved in N metabolism for these organisms. However, it is known that most dinoflagellates species with permanent chloroplasts can live in medium supplemented strictly with various inorganic nutrients, nitrate (${NO}_{3}^{-}$) being the predominant N form (Hansen, 2011). This implies that N assimilation genes are present in these species and, because this process is remarkably well conserved in plants and algae, it is likely that parallels can be made with dinoflagellates (**Figure 1**, see further sections for details). This review will first address these parallels by describing what is currently reported about the molecular components involved in N metabolism in plants and eukaryotic algae. We will then discuss the particular case of mixotrophy for the uptake of N in dinoflagellates. This nutrient being essential for synthesis of amino acids, nucleic acids, chlorophylls, and toxins, it is a major factor limiting growth. In conditions of N-stress dinoflagellate cells either die or modify their metabolism and trophic behavior to ensure their survival. We will finish by presenting the various adaptations used by dinoflagellates to cope with N stress. Throughout the text, we will compare the dinoflagellate N responses to those of diatoms to examine which environmental conditions could favor one group of organisms over the other.

## OVERVIEW OF THE MARINE N CYCLE

The marine N cycle is probably the most complex of the biogeochemical cycles, as it involves various chemical forms and multiple transformations that connect all marine organisms. In this section and the following one, we will begin by giving a brief overview of the N cycle to better understand now N flux in the oceans and what chemical forms are the most relevant for dinoflagellates. We will then describe the molecular mechanisms of N transport in plants and algae. We will finish with what is currently known about physiological N uptake in dinoflagellates and how our understanding has been helped by genomic and functional studies in diatoms.

About 94% of the oceanic N inventory exists as biologically unavailable dissolved nitrogen gas (N_2_; Gruber, 2008). This gas can be made bioavailable through N_2_-fixation, a process carried out by photoautotrophic prokaryotes, mainly cyanobacteria, using iron-dependent nitrogenases to catalyze reduction of N_2_ to ${NH}_{4}^{+}$. N_2_-fixation thus provides a counterbalance to the loss of bioavailable N through denitrification (${NO}_{3}^{-}$ to N_2_) and anaerobic ammonium oxidation (Annamox, ${NH}_{4}^{+}$ to N_2_), both of which are anaerobic reactions catalyzed by bacteria. The 6% of biologically available N exists primarily as ${NO}_{3}^{-}$ (~88%) and dissolved organic nitrogen (DON, ~11.7%; Gruber, 2008). The remaining 0.3% is found in other chemical forms, such as ${NO}_{2}^{-}$, ${NH}_{4}^{+}$, nitrous oxide (N_2_O) and particulate organic nitrogen (PON; Gruber, 2008).

The distribution and composition of fixed N forms vary with depth as direct consequences of combined biological and physical processes. In the euphotic zone, C fixation by photosynthesis drives the assimilation of inorganic N in order to sustain growth. If the resulting organic N produced is released into the seawater in the euphotic zone, most can be directly reassimilated, remineralized into inorganic N or respired for energy production. However, some organic N will sink down to the aphotic zone where ammonification and nitrification will remineralize it back to inorganic N. Finally, ocean circulation and mixing can return this remineralized N to the euphotic zone where it can be used to sustain new growth. The direct consequences of this biogeochemical loop are that surface waters are generally depleted in inorganic N while the deep oceans are enriched (Gruber, 2008). Conversely, concentrations of PON and DON are much higher near the surface than deeper in the oceans (Gruber, 2008). While this scenario holds in the open ocean, anthropogenically derived N additions have significantly changed the nutrient states in the coastal regions of the world (Rabalais, 2002; Beman et al., 2005). Agricultural runoffs and aquaculture industries bring sizeable amounts of new inorganic and organic N which tend to accelerate the N cycle in coastal ecosystems (Galloway et al., 2004; Beman et al., 2005; Gruber, 2008; San Diego-McGlone et al., 2008). The main engine driving this accelerated N cycle is the ability of phytoplanktonic species to take up N directly from the environment using very efficient transport systems.

## UPTAKE OF NITROGEN USING TRANSPORTERS

The work of Epstein and Hagen on ionic transport in plant rhizodermal cells was among the first to describe the dynamics of transporters (Epstein and Hagen, 1952). They found that the kinetics of ion uptake shared all the characteristics of classic Michaelis-Menten enzyme catalyzed reactions, although uptake of potassium (K) and rubidium was later shown to have a low *K*_m_ or a high *K*_m_ depending if the external ionic concentrations were low (μM) or high (mM), respectively (Epstein et al., 1963). In plants and eukaryotic algae, physiological import of inorganic and organic N is also generally dependent on environmental concentrations (Doddema and Telkamp, 1979; Franco et al., 1988; Wilson and Walker, 1988; Siddiqi et al., 1990; Galvan et al., 1996; Howitt and Udvardi, 2000; Rentsch et al., 2007; Wang et al., 2008). Expression of transport proteins in heterologous systems such as yeast and *Xenopus* oocytes has greatly helped to determine their biochemical properties (Galvan and Fernandez, 2001; Rentsch et al., 2007). At the molecular level, dual affinity can be explained by the presence of a group of transporters, which individually have either a high or a low affinity for their substrates. However, dual affinity can also result when an individual transporter is able to switch between the two affinities. The best example of a switching transporter is CHL1/AtNRT1.1 in *Arabidopsis*, where phosphorylation of threonine 101 (T101) changes its activity from a low-affinity to a high-affinity ${NO}_{3}^{-}$ transporter (Liu and Tsay, 2003). Interestingly, T101 was also shown to be involved in ${NO}_{3}^{-}$ sensing, as assessed by the ability of ${NO}_{3}^{-}$ to induce expression of genes involved in ${NO}_{3}^{-}$ metabolism. Mutants mimicking the phosphorylated form of the transporter were unable to elicit a low-affinity ${NO}_{3}^{-}$ response, whereas mutants mimicking the dephosphorylated form had an increased ${NO}_{3}^{-}$ response at all concentrations of N (Ho et al., 2009). CHL1/AtNRT1.1 was thus named a “transceptor.” Finally, a last group of transporters is made up of channel-like proteins such as the major intrinsic proteins (MIPs), a family which also contains aquaporins. MIPs provide facilitated diffusion of ${NH}_{4}^{+}$ and urea into plant cells and tonoplasts and have only low affinity for their substrates (Gaspar et al., 2003; Liu et al., 2003b; Jahn et al., 2004; Loque et al., 2005).

Nitrogen transporters and channels are often multi-selective and differentially regulated. For example, the *Chlamydomonas reinhardtii* transporters CrNRT2.1 and CrNRT2.3 transport both ${NO}_{3}^{-}$ and ${NO}_{2}^{-}$ with identical or different affinities, respectively. CrNRT2.1 has a high affinity for both ${NO}_{3}^{-}$ and ${NO}_{2}^{-}$, while CrNRT2.3 has a low affinity for ${NO}_{3}^{-}$ and a high affinity for ${NO}_{2}^{-}$ (Fernandez and Galvan, 2007). Generally, however, most transporters of the same family or subfamily share similar substrate selectivity and affinity. As for the regulation patterns, some transporters are constitutively expressed while others are influenced by different conditions such as pH, light and level of substrates in the environment.

Facilitated diffusion such as carried out by MIPs follows down the concentration gradient and thus has no requirement for energy. However, for the majority of inorganic and organic N compounds, transport goes against a concentration gradient and thus needs a source of energy. Plants and algae produce a proton motive force (PMF) using H^+^-ATPases at their plasma membranes and other cell compartments. The PMF is an electrochemical gradient exploited by transporters, such as those for ${NO}_{3}^{-}$, ${NO}_{2}^{-}$, the high-affinity system of urea, some amino acids and peptides. These transporters work either by the symport or antiport of H^+^, and their activities are thus dependent on pH (Galvan and Fernandez, 2001; Liu et al., 2003a; De Angeli et al., 2006; Fernandez and Galvan, 2007; Rentsch et al., 2007; Sugiura et al., 2007). Others, such as the high-affinity ${NH}_{4}^{+}$ uniporter AMT1.1 from *Arabidopsis*, exploit the electrical gradient generated by the PMF, and thus do not require H^+^ transport (Ninnemann et al., 1994; Howitt and Udvardi, 2000).

One problem concerning the use of a PMF in marine organisms is that seawater is typically alkaline (pH ≥ 7.8). Fortunately, seawater also contains abundant sodium (Na^+^, 450–500 mM), and as in animal cells, many marine algae can exploit these Na^+^ gradients for uptake of nutrients such as ${NO}_{3}^{-}$, ${PO}_{4}^{3-}$, glucose, amino acids and silica (Hellebust, 1978; Bhattacharyya and Volcani, 1980; Raven and Smith, 1980; Boyd and Gradmann, 1999). Moreover, existence of P-type Na^+^-ATPases in marine algae was confirmed in *Tetraselmis viridis* and *Heterosigma akashiwo* (Gimmler, 2000). The Na^+^ versus H^+^ powered-transport in, respectively, marine and freshwater/terrestrial organisms, is however not an absolute rule. For example, the freshwater chlorophyte *Ankistrodesmus braunii* requires Na^+^ for the transport of ${PO}_{4}^{3-}$, while Charales living in brackish waters typically use H^+^ symporters for nutrient transport (Ullrich and Glaser, 1982; Bisson et al., 2006). The strong negative membrane potential of characean cells is thought to enable H^+^-symport even in alkaline environments (Bisson et al., 2006). Notably, Charales are also able to use Na^+^-coupled transport for ${PO}_{4}^{3-}$, urea and Cl^-^ (Sanders et al., 1985; Walker et al., 1993; Reid et al., 2000). To date, no comparisons of the energetics of transport have been made between dinoflagellates living in freshwater and those living in seawater.

Physiological uptake of ${NO}_{3}^{-}$, ${NO}_{2}^{-}$, ${NH}_{4}^{+}$, urea and other DON by chloroplast-containing dinoflagellates has been reported in both field and laboratory studies (Harrison, 1976; Kudela and Cochlan, 2000; Fan et al., 2003; Leong and Taguchi, 2004; Collos et al., 2007; Maguer et al., 2007; Lee, 2008; Loureiro et al., 2009; Leong et al., 2010; Pernice et al., 2012). Most describe the uptake kinetics in relation to cell growth, and variability in the kinetics has emerged as an important feature. Depending on the experimental sampling conditions, different intraspecific half-saturation constant (*K*_m_) values for ${NO}_{3}^{-}$, ${NH}_{4}^{+}$ and urea were reported in *Lingulodinium polyedrum* and *Alexandrium catenella* (Smayda, 1997; Kudela and Cochlan, 2000; Collos et al., 2007). In the latter species, *K*_m_ for ${NH}_{4}^{+}$ and *K*_m_ for urea varied from 0.2 to 20 μM and 0.1 to 44 μM over a 4-year period, respectively (Collos et al., 2007). In the same study, the authors’ measured *K*_m_ values range from 0.5 to 6.2 μM ${NH}_{4}^{+}$ within a 3-day interval, showing how fast transport kinetics can change within the same dinoflagellate population. Similar variations were also noted in *L. polyedrum* (Harrison, 1976). In a 2005 review, Collos et al. (2005) noted a linear relationship between *K*_m_ for ${NO}_{3}^{-}$ and ambient ${NO}_{3}^{-}$ concentrations for various freshwater and marine unicellular algae in the field. They proposed that most phytoplankton possess an ability to physiologically acclimate to different ${NO}_{3}^{-}$ concentrations. The variations of *K*_m_ observed for ${NH}_{4}^{+}$ and urea in dinoflagellates suggest that acclimation could be generalized to various N forms, not only ${NO}_{3}^{-}$. From a molecular perspective, these results suggest that different combinations of transporters each with particular kinetics and level of expression/activity will be found in dinoflagellates.

Generally, when growing in presence of various different N compounds, dinoflagellates (as well as plants and algae) prefer to take up ${NH}_{4}^{+}$. However, there is a concentration threshold above which ${NH}_{4}^{+}$ becomes toxic to the cells, and this threshold seems to be species-specific. For example, in *A. minutum*, ${NH}_{4}^{+}$ concentrations of 25 μM and higher lead to growth inhibition while for *A. tamarense* and *Cochlodinium polykrikoides*, this threshold was not observed until 50 μM (Chang and McClean, 1997; Leong and Taguchi, 2004; Lee, 2008). Another tendency in dinoflagellates is inhibition of ${NO}_{3}^{-}$ uptake when in the presence of ${NH}_{4}^{+}$. In *A. minutum*, this inhibition was found to be greater when the cells were in N-sufficient compared to N-deprived conditions (Maguer et al., 2007). This suggests that when N is limiting, uptake of different forms will be favored over strict assimilation of ${NH}_{4}^{+}$ which has a reduced energy cost. Curiously, different blooming populations of dinoflagellates were found to have high uptake rates for urea and/or amino acids, and these rates were always higher than the rates for ${NO}_{3}^{-}$ uptake (Kudela and Cochlan, 2000; Fan et al., 2003; Collos et al., 2007). In *L. polyedrum*, the urea uptake rate was also about 2 times more than that of ${NH}_{4}^{+}$, even if environmental urea concentrations were less than ${NH}_{4}^{+}$ (Kudela and Cochlan, 2000). Taken together, these observations suggest that dinoflagellates possess a full suite of transporters for inorganic N and organic N forms, that they have the biochemical means to assimilate these N forms, and that they show a great physiological plasticity in response to external N types and concentrations.

Dinoflagellates are able to store large amounts of inorganic and organic N forms. Comparison of N uptake and assimilation rates at various growth rates in *A. minutum* showed that most of the ${NO}_{3}^{-}$ and ${NH}_{4}^{+}$ taken up in 1 h was not assimilated, and it was hypothesized that the unassimilated N was stored within the cell (Maguer et al., 2007). This species was also found to have a great storage capacity for amino acids (Flynn et al., 1996), and a similar storage capability was also described in *A, catenella*, *A. tamarense*, and *Amphidinium cartarae* as well as other unicellular algae (Dortch et al., 1984; Collos et al., 2004; Fauchot et al., 2005). One recent study, using Nanoscale Secondary-Ion Mass Spectrometry (NanoSIMS) and transmission electron microscopy, showed that *Symbiodinium* spp*.* temporarily stored N in the form of uric acid crystals after sudden increases in environmental N (Kopp et al., 2013). Indeed, pulses of ^15^N-labeled ${NH}_{4}^{+}$, ${NO}_{3}^{-}$ or aspartic acid promoted accumulation of cytosolic crystalline uric acid inclusions in the zooxanthellae, which were formed in only 45 min in the case of ${NH}_{4}^{+}$. After 24 h of chasing with unlabeled-${NH}_{4}^{+}$ seawater, the inclusions completely dissolved and were remobilized uniformly within the cell. These results suggest that dinoflagellates might store their N within the cytosol, in contrast to plants where up to 50 mM of ${NO}_{3}^{-}$ can be stored in the vacuoles (Cookson et al., 2005). The chemical nature of the long-term storage of N in dinoflagellates is still unclear.

Another interesting feature of N transport in dinoflagellates is the ability of some species to take up substantial amounts of various N forms in the darkness (**Table 1**). Dinoflagellates often display a diurnal vertical migration (DVM) in the water column and, because ${NO}_{3}^{-}$ concentrations increase with depth, dark ${NO}_{3}^{-}$ uptake was first described as a means to sustain uninterrupted growth by meeting their N requirements under conditions where the cells cannot photosynthesize (Harrison, 1976). It was further suggested that the DVM of dinoflagellates gave them a competitive advantage for N uptake over the non-motile diatoms (Harrison, 1976; Smayda, 1997). Paasche et al. (1984) showed that uptake efficiency and the N form preferentially taken up in the dark were species-specific. At one end of the spectrum, *P. minimum* took up ${NH}_{4}^{+}$ and ${NO}_{3}^{-}$ at similar rates in the light or in the dark, while at the other end, *Gyrodinium aureolum (Hulburt)* in the dark had smaller rates of uptake for ${NH}_{4}^{+}$ and did not take up ${NO}_{3}^{-}$ in N-sufficient conditions (Paasche et al., 1984). Recently, dark uptake of ${NO}_{3}^{-}$, ${NH}_{4}^{+}$ and urea was confirmed in *A. tamarense* (Leong et al., 2010).

**Table 1:** Comparison of adaptation mechanisms to N stress between dinoflagellates and diatoms.

Mechanism	Dinoflagellates	Diatoms
Encystment	✓	✓
C storage	✓	✓
High-affinity transporters	✓	✓
Decreased Internal N pool	✓	✓
Ornithine-urea cycle	✓	✓
Symbiosis	✓	✓
Transcriptional control	✓	✓
Circadian control	✓	✗
Mixotrophy	✓	✗
Vertical migration/dark nitrate uptake	✓	✗

In contrast to dinoflagellates where molecular characteristics of transporters are mainly based on predictions from physiological studies, the three presently available diatom genomes have helped considerably to better appreciate the full extent of N transport and assimilation in these organisms (Armbrust et al., 2004; Bowler et al., 2008; Lommer et al., 2012). Consistent with their fast growth rate and high productivity, diatom genomes were found to contain multiple transporters for ${NO}_{3}^{-}$, ${NH}_{4}^{+}$, urea and other organic N forms (Armbrust et al., 2004; Bender et al., 2012). Analysis of genomes and studies in *Cylindrotheca fusiformis* revealed that diatoms seemed to possess twice as many transporters for ${NH}_{4}^{+}$ compared to ${NO}_{3}^{-}$ (Hildebrand and Dahlin, 2000; Allen, 2005; Hildebrand, 2005). It was suggested that these numbers reflect the fact that marine phytoplankton generally face low concentrations of ${NH}_{4}^{+}$, while ${NO}_{3}^{-}$ concentrations are higher (Allen, 2005). Amino acid sequence analysis of five ${NH}_{4}^{+}$ transporters of *C. fusiformis* showed that they shared 40% similarity with the vascular plant ${NH}_{4}^{+}$ transporters AMT1 and AMT2 (Hildebrand, 2005). Furthermore, rescue by functional complementation of a yeast strain missing all three of its native ${NH}_{4}^{+}$ transporters not only confirmed the functionality of the identified transporters, but also showed that AMT1 in diatoms were much more efficient transporters than AMT2 (Hildebrand, 2005). Identification and characterization of ${NO}_{3}^{-}$ transporter sequences were also made in C*. fusiformis* (Hildebrand and Dahlin, 2000). As a general rule, genomic data and functional characterization showed that marine N transporters share sequence homology with N transporters of terrestrial and freshwater organisms, and that these tools can be used to better understand the responses of an organism to different N forms and concentrations. In dinoflagellates, the immense sizes of their genomes and the high gene copy number have long hindered sequencing projects. However, efforts are now being made to sequence the smallest of dinoflagellate genome, *Symbiodinium* (Shoguchi et al., 2013). There is no doubt that the presently available transcriptomic data for *Alexandrium*, *Karenia*, *Lingulodinium,* and *Symbiodinium*, as well as the upcoming genome sequences will help in unraveling the complexity of N transport and assimilation in dinoflagellates (Morey et al., 2011; Bayer et al., 2012; Beauchemin et al., 2012; Hackett et al., 2013).

## UPTAKE OF NITROGEN BY FEEDING

Because “food” comes from whole organisms (or parts of them), it contains a large spectrum of inorganic and organic matter, in which each individual compound is not ingested differently. For this reason, the following section will begin by describing general feeding mechanisms measured in mixotrophic studies of dinoflagellates, taking for granted that N is not ingested differentially than from other nutrients. Following this, we will discuss some specific examples of the N contribution obtained from feeding and of the inorganic N influence on the mixotrophic behavior of different dinoflagellates. We will finish with a model proposed by Jeong et al. (2010) where mixotrophy explains the outbreak and persistence of HABs in aquatic ecosystems limited in inorganic nutrients.

Mixotrophic dinoflagellates (MTDs) and heterotrophic dinoflagellates (HTDs) have similar feeding strategies. These strategies include (1) raptorial feeding, where the predator actively searches for its prey, (2) filter/interception, where the predator generates feeding currents to drag the prey into proximity of its feeding parts, and (3) diffusion feeding, where the predator passively wait until the prey comes close (Fenchel, 1987; Jeong et al., 2010). Raptorial feeding uses 3 mechanisms for ingestion of preys, (1) direct engulfment (phagocytosis), (2) pallium feeding, where a feeding veil is deployed around the prey, and (3) peduncle feeding, in which a straw-like structure is used to siphon out the prey’s cytoplasm (Jacobson, 1987; Jeong et al., 2010). Pallium feeding has still not been observed in MTDs, and thus seems to be unique to HTDs. HTDs are also able to engulf bigger preys than MTDs. In fact, the upper size limit of prey is generally proportional to the size of MTDs, while this correlation is not observed for HTDs (Jeong et al., 2005b). It was suggested that morphological adaptations to the sulcus, the “mouth” of most HTDs, as well as a stronger pulling force for ingestion of prey, could enable them to ingest larger prey (Jeong et al., 2010). In general, the prey upper size limits are greater when using pallium and peduncle feeding than when using engulfment. Spectacularly, some pallium and peduncle feeders are able to ingest prey up to 10 times their size (Jacobson, 1987). As for prey types, MTDs and HTDs feed on a wide array of taxa. They were shown to ingest cryptophytes, haptophytes, chlorophytes, prasiophytes, raphidophytes, diatoms, heterotrophic nanoflagellates, ciliates, and other dinoflagellates (Jacobson and Anderson, 1986; Hansen, 1991; Bockstahler and Coats, 1993b; Strom and Buskey, 1993; Nakamura et al., 1995; Tillmann, 2004; Jeong et al., 2005a, 2008; Menden-Deuer et al., 2005; Adolf et al., 2007; Berge et al., 2008). However, while some HTDs can feed on blood, flesh, eggs and early naupliar stages and adults forms of metazoans, no MTDs have been shown to do so (Miller and Belas, 2003; Parrow and Burkholder, 2004; Jeong et al., 2007).

In 1998, Stocker has proposed three physiological types of mixotrophic protists (Stoecker, 1998). Type III contains “photosynthetic” protozoa that require prey for growth and survival. In the same manner as HTDs, type III MTDs take the majority of their nutrients from feeding. Type I is made of “ideal” mixotrophs, which grow equally well either using light and inorganic nutrients or when they consume food. Very few MTDs with permanent chloroplasts are of this type. In fact, only 3 out of 36 reported species grew in the dark when prey was provided (Hansen, 2011). Most mixotrophs belong to type II, which was defined as “phagotrophic” algae that primarily photosynthesize and can assimilate inorganic nutrients. In this group, different proportions of N can be taken up either by transporters or through feeding. Estimations of these proportions have been made using *in situ* grazing experiments in *Ceratium furca* and *Akashiwo sanguinea* (Bockstahler and Coats, 1993a; Smalley and Coats, 2002). In *C. furca*, hourly ingestion rates (I) of the fluorescently labeled prey *Strobilidium* spp., were measured by dividing the mean number of food vacuoles in the predator with the incubation time of prey and predator. The % body N was then estimated as daily ingestion relative to cellular N content of *C. furca* [100 × 24 h × I × (cellular N of prey/cellular N *C. furca*)]. On average, the % N obtained through feeding was 6.5 % with a maximal value of 51 % (Smalley and Coats, 2002). Similarly in *A. sanguinea*, a gut clearance/gut fullness approach estimated an average of 4 % N obtained through feeding with a maximal value of 18.5 % (Bockstahler and Coats, 1993a). Both of these species have low averages, which signifies that under low light and with inorganic nutrient concentrations normally found in the environment, uptake of N by transporters is dominant over feeding. Interestingly, *C. furca* and *A. sanguinea* show only a marginal increase in growth rate with increasing prey concentrations (Hansen, 2011). However, in another 6 out of 36 chloroplast-containing species, a large increase in growth rates with increasing prey concentrations was observed (Hansen, 2011). Although the % body N in these species was not estimated, it could be argued that a larger proportion of N was obtained through feeding compared to *C. furca* or *A. sanguinea*. An extreme case of mixotrophy is observed in some dinoflagellates such as the *Dinophysis* genera that may contain chloroplasts of cryptophyte, haptophyte or cyanobacterial origin (Qiu et al., 2011). All the species studied in this genus cannot grow on an inorganic medium alone, as all require both light and prey for growth and survival (Park et al., 2006; Kim et al., 2008; Nishitani et al., 2008a,b; Garcia-Cuetos et al., 2010; Hansen, 2011). *Dinophysis* spp. are thus considered obligate mixotrophs. It is still unclear what are the proportions of N obtained by feeding and by transporters in these organisms.

The grazing behavior of some MTDs was shown to be under the influence of light and inorganic nutrient concentrations. In laboratory cultures of *Gyrodinium galatheanum* and *C. furca*, the incidence of feeding was negatively correlated with the amount of inorganic N present in the medium (Li et al., 2000; Smalley et al., 2003). In *C. furca*, cells only started to ingest food after 11 to 16 days in ${NO}_{3}^{-}$ -depleted medium (Smalley et al., 2003). Similarly in *P. minimum*, additions of ${NO}_{3}^{-}$ inhibited feeding (Stoecker et al., 1997). Curiously, this organism followed a diel pattern of prey ingestion with peaks in the afternoon and evening, and a trough in the morning. Moreover, the number of food vacuoles observed within *P. minimum* cells did not change between surface populations and the ones deeper in the water column. Because of these spatio-temporal evidences, the authors suggested that feeding in this organism was primarily a means for obtaining limiting nutrients, and not a mechanism to supplement C nutrition during light limitation (Stoecker et al., 1997). In contrast to the above-mentioned species, prey ingestion rates in *Fragilidium cf. mexicanum* was shown to be independent of inorganic N concentrations (Jeong et al., 1999). Also, most of the chloroplast-containing MTDs were reported to ingest prey in N-replete conditions (Hansen, 2011). Thus, the effects of inorganic N on grazing behavior must be species-specific.

It was long believed that bacteria were too small to be ingested by dinoflagellates. In the last few years, however, fluorescence and transmission electron microscopy observations revealed that multiple HTDs and MTDs were able to feed on heterotrophic bacteria and cyanobacteria (Jeong et al., 2005a, 2008; Seong et al., 2006; Glibert et al., 2009). In particular, feeding on the N_2_-fixing *Synechococcus* spp. was seen in 18 species reported to form HABs (Jeong et al., 2005a; Seong et al., 2006; Glibert et al., 2009). Generally, when prey concentration was high (10^6^ cells/ml), the ingestion rates increased with increasing size of the dinoflagellate predators (Jeong et al., 2005a). Moreover, ingestion rates of *Synechococcus* were comparable to those observed in heterotrophic nanoflagellates (Seong et al., 2006). A mixture of *P. mininum* and *P. donghaiense* was able to remove up to 98% of the *Synechococcus* population within 1 h, showing that grazing by these species on bacteria could be very substantial (Jeong et al., 2005a). Thus, bacterivory in dinoflagellates was suggested to be a cause of HABs outbreaks and persistence in nutrient-limited waters (Glibert et al., 2009; Jeong et al., 2010). A model was further proposed where MTDs would supply their N requirement by ingesting cyanobacteria, while meeting their P requirement by ingesting heterotrophic bacteria, which are reported to generally have a high P:N ratio (Jeong et al., 2010). This model as yet to be tested in the environment.

## NITROGEN ASSIMILATION AND METABOLISM

Once inorganic N has entered the cells, phototrophic and MTDs can either store it or assimilate it in the form of amino acids. Whether organic N is taken up by means of transport or by ingestion of food, all dinoflagellates must metabolize it for further use using enzymes such as ureases, hydrolases, peptidases, and aminotransferases (also known as transaminases). In this section, we will start by giving a brief description of the enzymes involved in inorganic N assimilation, based on the available knowledge from plants and algae. We will follow with different evidence on the presence of these enzymes in dinoflagellates, particularly the NR of *L. polyedrum*, which was found to be under the control of a circadian clock. We will finish with a description of N assimilation in diatoms with emphasis on the newly discovered ornithine-urea cycle (OUC).

There are two structurally different types of NR, dissimilatory and assimilatory. The first type is found mainly in anaerobic prokaryotes, but is also present in some eukaryotes, such as the benthic foraminiferans and some fungi (Shoun and Tanimoto, 1991; Usuda et al., 1995; Risgaard-Petersen et al., 2006). These organisms use their dissimilatory NR to respire ${NO}_{3}^{-}$ instead of O_2_. Direct reduction of ${NO}_{3}^{-}$ to ${NH}_{4}^{+}$ is a process named dissimilatory ${NO}_{3}^{-}$ reduction to ammonium (DNRA), and is well documented in prokaryotes such as large sulfur bacteria (Preisler et al., 2007). Surprisingly, DNRA was also identified in the benthic diatom *Amphora coffeaeformis* (Kamp et al., 2011). The authors suggested that this organism respired ${NO}_{3}^{-}$ as a means to survive dark and anoxic conditions. Considering that some dinoflagellates can form resting cysts that will sink in the benthic zone, and that others are also living there, dinoflagellates might also rely on the energy produced by dissimilatory NRs for excystment and for survival. However, the second type, assimilatory NR (usually just called NR) is the most common form in both eukaryotes and prokaryotes and is the type that was implied until now in this review. It catalyzes the reduction of ${NO}_{3}^{-}$ to ${NO}_{2}^{-}$ for assimilation. NRs usually form homodimers, but homotetramers have been reported in organisms such as the green algae *Chlorella* (Howard and Solomonson, 1982). Each monomer contains an electron transport chain made of three prosthetic groups: a flavine adenine dinucleotide (FAD), a cytochrome b557 and a molybdenum cofactor (Guerrero et al., 2003; Heldt and Piechulla, 2011). These groups are ubiquitous in all reported NR and are necessary for the stepwise transfer of 2 electrons from NAD(P)H to ${NO}_{3}^{-}$ in eukaryotes. In contrast, prokaryotes use ferredoxins (Fd) or NADH for reduction of ${NO}_{3}^{-}$ and their NR is generally membrane-bound whereas the eukaryotic one is soluble (Moreno-Vivian et al., 1999; Guerrero et al., 2003). Eukaryotic NRs are generally localized to the cytoplasm.

In photosynthetic eukaryotes, once ${NO}_{3}^{-}$ has been reduced, the resulting ${NO}_{2}^{-}$ is imported into the chloroplasts where it will be further reduced. There are two types of assimilatory NiR. The first one uses reduced-Fd from the photosynthetic transport chain as an electron donor, while the second one, usually found in non-photosynthetic organisms, uses NAD(P)H (Guerrero et al., 2003). Both types require six electrons to catalyze the reduction of ${NO}_{2}^{-}$ to ${NH}_{4}^{+}$. Similarly to NR, Fd-NiRs contain three prosthetic groups: an iron-sulfur center, a FAD and a siroheme (Guerrero et al., 2003; Heldt and Piechulla, 2011). Most of NiRs are soluble and work as individual enzymes. Generally, NiRs have a greater affinity for ${NO}_{2}^{-}$ than NRs have for ${NO}_{3}^{-}$ (Guerrero et al., 2003). As a consequence, ${NO}_{2}^{-}$ is completely reduced to ${NH}_{4}^{+}$ and does not accumulate within the cells. In plants and algae, there are GS localized to the chloroplast and the cytosol (Chen and Silflow, 1996; Fernandez and Galvan, 2007; Forde and Lea, 2007). GS adds ${NH}_{4}^{+}$ to Glu using ATP to produce 1 molecule of Gln. Gln is a central metabolite, which acts as a precursor for the biosynthesis of purines, pyrimidines, proteins, and ureides (Forde and Lea, 2007). GOGATs are found exclusively in the chloroplasts/plastids in plants and algae (Fernandez and Galvan, 2007; Forde and Lea, 2007). They combine Gln to 2-oxoglutarate using 2 electrons from either reduced-Fd or NAD(P)H and produce 2 molecules of Glu. The α-amino group of Glu can then be transferred by amidotransferases to a wide variety of 2-oxo acid acceptors to form amino acids, and back to form Glu, when amino acids and 2-oxoglutatare are abundant. GDH present in the mitochondria can also use ${NH}_{4}^{+}$ and 2-oxoglutarate to produce Glu. Thus, Glu and Gln are at the crossroads of amino acid metabolism where they act both as N acceptors and N donors.

The presence of all inorganic N assimilation enzymes was tested indirectly or directly in some MTDs. In *Symbiodinium*, analysis of transcriptomic data revealed sequences for NR and NiR (Leggat et al., 2007; Bayer et al., 2012). Moreover, NanoSIMS observations showed that after pulses of ^15^N-labeled ${NO}_{3}^{-}$, zooxanthellae assimilated this N form before translocation to the coral host (Kopp et al., 2013). Because the host does not encode any NR or NiR genes, it was concluded that the dinoflagellate symbionts were responsible for the assimilation. In dinoflagellates, it is believed that N is principally assimilated by the GS-GOGAT pathway. In fact, GS activities were directly measured in *S*. *microadriaticum* and the use of the GS inhibitor L-methionine sulfoximine (MSX) strongly reduced the uptake of ${NH}_{4}^{+}$ (Anderson and Burris, 1987). Use of the GOGAT inhibitor azaserine also led to inhibition of ${NH}_{4}^{+}$ uptake (Rahav et al., 1989). Moreover, 1 h pulses in ${NH}_{4}^{+}$-enriched seawater led to a relative ~2 fold increase in levels of Gln and Glu, followed by a decrease back to control levels after a 3 h chase with normal seawater (Pernice et al., 2012). Increases in levels of Gln and Glu were also reported when pulses of ^15^N-labeled ${NH}_{4}^{+}$, ${NO}_{3}^{-}$ or urea were applied (Summons and Osmond, 1981). In *Karenia brevis*, a microarray-analysis of N-depleted cultures revealed that sequences for GS and transporters of ${NO}_{3}^{-}$ and ${NH}_{4}^{+}$ were upregulated compared to N-replete cultures (Morey et al., 2011). In *L. polyedrum*, a NR was purified by affinity chromatography and its activity was measured using ${NO}_{3}^{-}$ as substrate (Ramalho et al., 1995). Antibodies were then raised against the protein in order to measure the daily level of expression. Interestingly, it was found that both NR activity and the amount of the protein oscillated under light-dark and constant light conditions, the latter being a hallmark of circadian rhythms (Ramalho et al., 1995). Furthermore, it was reported in this organism that ${NO}_{3}^{-}$ deficiency shortened the period of the circadian rhythms of bioluminescence and photosynthesis. MSX added to cultures containing a saturating amount of ${NO}_{3}^{-}$ mimicked the effects of ${NO}_{3}^{-}$ deficiency on circadian period, arguing for the existence of GS. Altogether, these experiments strongly suggest MTDs possess the full suite of enzymes required for N assimilation and that these enzymes are homologous to those of other photosynthetic eukaryotes.

Circadian clocks confer a selective advantage to organisms by preparing their internal biochemistry for upcoming rhythmic environmental cues, the most typical of these being light and darkness produced the succession of days and nights. These rhythmic cues are called *zeitgebers* (time givers), because an organism’s circadian clock can entrain or synchronize to it. The fact that ${NO}_{3}^{-}$-deficiency or ${NO}_{3}^{-}$ pulses act on the phase and the period of the endogenous clock of *L. polyedrum* means that ${NO}_{3}^{-}$ acts as a non-photic *zeitgeber* (Roenneberg and Rehman, 1996). It also indicates that the internal clock can react adaptively to changes in ${NO}_{3}^{-}$ concentration. Usually the greatest responses of a *zeitgeber* happen during the times when the organism is least likely to receive a cue, for example by light during the night. *L. polyedrum* swims at the surface during the day, but in the late afternoon sinks in the water column to where ${NO}_{3}^{-}$ concentrations are higher. Thus, the effects of ${NO}_{3}^{-}$ pulses are greatest early at dawn where the organisms must optimize between taking and assimilating more ${NO}_{3}^{-}$ or photosynthesizing. However, these effects on the clock must be interpreted carefully since the greatest uptake rates of ${NO}_{3}^{-}$ or ${NH}_{4}^{+}$ in all dinoflagellate species tested, including *L. polyedrum*, were obtained during the middle of the light period rather than the night (Harrison, 1976; Dortch and Maske, 1982; Paasche et al., 1984). This suggests that the C skeletons and electrons provided by photosynthesis are more important factors affecting the efficiency of uptake than is the N abundance alone. Furthermore, because the circadian clock also controls the activity and expression of the NR *in L. polyedrum*, it means the N metabolism in this species is both involved in the inputs and the outputs of the clock. Circadian-control of the NiR activity in *C. reinhardtii* and on the expression of the NR gene, *NIA2*, in *Arabidopsis thaliana* has also been reported (Pilgrim et al., 1993; Iliev et al., 2006). Thus, circadian regulation of the N metabolism in MTDs might be more common than what is currently appreciated.

**FIGURE 2:**
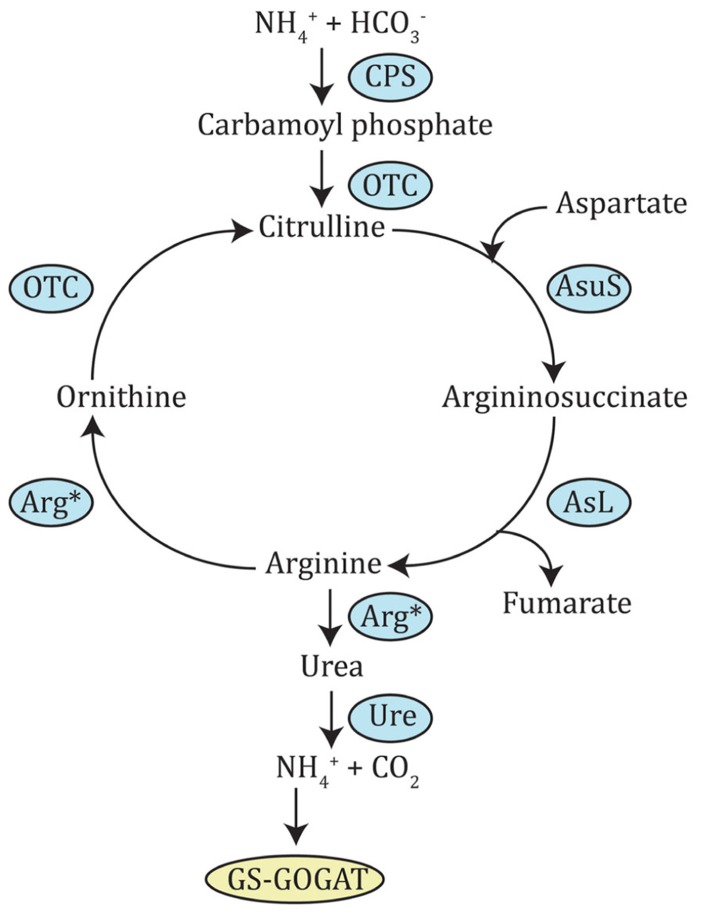
**Ornithine-urea cycle.** This pathway is derived from that identified in diatoms. CPS, carbamoyl phosphate synthase; OTC, ornithine carbamoyltransferase; AsuS, argininosuccinate synthase; AsL, argininosuccinate lyase; Arg*, arginase (depicted twice, because it produces both ornithine and urea); Ure, urease.

All the enzymes needed for N assimilation have been identified in diatoms by analysis of their genome sequence (Armbrust et al., 2004; Bowler et al., 2008; Lommer et al., 2012). Surprisingly, the genome also encoded all enzymes required for the OUC, present in metazoans but absent in plants and green algae (Armbrust et al., 2004; Allen et al., 2011). This cycle seems to be fully integrated within diatom metabolism, principally through ornithine and arginine intermediates. Ornithine is a precursor for the synthesis of polyamines, which, among other functions, are necessary for the precipitation of silica during frustule formation, in a reaction catalyzed by ornithine decarboxylase (Armbrust et al., 2004). Ornithine can also be directly converted to proline by ornithine cyclodeaminase, making this intermediate an entry point into amino acid metabolism. As for arginine, it is used by nitric oxide synthase to synthesize nitric oxide, an important signaling molecule in plants and animals (Gupta et al., 2011). A pathway leading to the formation of the energy-storage molecule creatine-phosphate, was also found to originate from arginine (Armbrust et al., 2004). Integration of the OUC with the TCA cycle and the GS-GOGAT pathway (see next section) were also shown in *Phaeodactylum tricornutum* (Allen et al., 2011). In order to check if dinoflagellates could also possess all the genes necessary for the OUC, BLAST searches against dinoflagellate nucleotide databases were performed using the protein sequences of *T. pseudonana* and *P. tricornutum* as queries. In fact, *Alexandrium tamarense* had candidates for all OUC enzymes (**Figure 2**, **Table 2**). This suggests dinoflagellates could also possess a complete OUC. In diatoms, OUC was recognized as an important N remobilization hub, and it has been suggested that it might give a general advantage when urea concentrations are high or any form of N are abundant in the environment. This was thought to perhaps explain the success and prevalence of diatoms over other phytoplankton species in eutrophic waters (Allen et al., 2011), but this idea may have to be revisited if dinoflagellates are also able to catalyze the same reactions.

**Table 2:** Nitrogen metabolizing enzymes similar to those in diatoms in the transcriptome of *Alexandrium tamarense.*

Enzyme	*E*-value	Query length (AA)	Coverage (%)	Accession
Carbamoyl phosphate synthase	0	1485	95	GAJB01000224
Ornithine carbamoyltransferase	e^-^^56^	352	90	GAIT01092113
Argininosuccinate synthase	e^-^^62^	418	95	GAIT01061622
Argininosuccinate lyase	e^-^^43^	469	78	GAJG01001449
Arginase	e^-^^18^	223	96	GAJB01022416
Urease	0	807	99	GAJB01002972

## ADAPTATIONS TO NITROGEN STRESS

Generally, dinoflagellates and diatoms need to cope with varying concentrations of N, particularly in the open ocean where it is often limiting. Thus, each group has developed various strategies, some of which are used by both, to respond and survive to oligotrophic environments (**Table 1**). In this section, we will present these responses based on environmental and laboratory experiments. We will discuss symbiosis and various physiological, behavioral and transcriptomic responses, with an emphasis on dinoflagellates.

Symbiosis with diazotrophs is an example of a strategy that is shared by some diatom and dinoflagellate species. The diatom genera *Hemiaulus* and *Rhizosolenia* both form endosymbiotic associations with the cyanobacteria *Richelia intracellularis* (Venrick, 1974; Carpenter et al., 1999). Both the hosts and the symbionts were observed to bloom together in the oligotrophic waters of the North Pacific Central Gyre and South West Atlantic Ocean. N_2_-fixation by *Richelia* introduced an amount of “new N” to the ecosystems that could even exceed the N_2_ fixed by non-blooming *Trichodesmium*. Carpenter et al. (1999) suggested that the silicate- and iron-enriched water of the Amazon River could have been factors in initiating and sustaining the blooms in the SW Atlantic Ocean. Silicate is required for the formation of the diatom frustule, while iron is necessary for the action of the diazotroph nitrogenases.

In N-limited conditions, endo- and ecto-symbiosis with cyanobacteria were exclusively identified within the dinoflagellate order Dinophysiales (Tarangkoon et al., 2010). In the genera *Amphisolenia* and *Triposolenia*, the symbionts were found inside the host, while the genera *Ornithocercus*, *Histioneis*, *Parahistioneis,* and *Citharistes* appeared to “farm” cultures of cyanobacteria outside the cells in an enlarged cingulum or in special cavities (Tarangkoon et al., 2010; Hansen, 2011). Further microscopic observations of *Ornithocercus* spp. suggested that they could also ingest their symbionts, depending on the size, shape, and color of the prey and on the presence of peduncle in the dinoflagellates. However, it is not clear if these species take up the majority of the external N fixed by the cyanobacteria using transporters and ingest the bacteria only occasionally, or if they “farm” the symbionts exclusively to feed on them. While it was not directly shown that *Symbiodinium* formed symbiotic association with cyanobacteria, the coral host was found to do so. In fact, whole communities of beneficial bacteria including N_2_-fixers and chitin decomposers were identified in all coral structures, including the surface mucous layer, tissue layers and the skeleton (Lesser et al., 2004; Rosenberg et al., 2007). Interestingly, amplification of the nitrogenase gene *nifH* in tissues of 3 different coral species revealed that 71% of the sequences came from a bacterial group closely related to rhizobia, the N_2_-fixers symbiotic with legumes (Lema et al., 2012). The products of N_2_-fixation were initially assimilated by the zooxanthellae, then translocated to the animal host, as determined by δ^15^N analysis (Lesser et al., 2007). Moreover, *Symbiodinium* population density was positively correlated with *nifH* sequence copy number, suggesting that growth and division of the zooxanthellae might be dependent upon the product of N_2_-fixation (Olson et al., 2009). Taken together, these examples suggest that the cyanobacteria barter their N_2_-fixing ability for protection and nutrients from their hosts, thus providing a selective advantage to the hosts in N-limited environments.

For the majority of diatom and dinoflagellate species, unable to form any symbiotic associations with diazotrophs, N-limitation sets in motion a series of physiological and behavioral modifications to avoid cell death. Generally, in both groups, N-limitation results in a decrease in the levels of proteins and chlorophylls (Lei and Lu, 2011; Hockin et al., 2012; Pleissner and Eriksen, 2012). Toxin content in *Alexandrium* sp. and *Ostreopsis ovata* was also shown to diminish when N-stressed (Wang and Hsieh, 2002; Leong et al., 2004; Hu et al., 2006; Vanucci et al., 2012). Furthermore, N-limitation or complete N-starvation induces a reduction or an arrest of the cell cycle in both groups (Lei and Lu, 2011; Morey et al., 2011; Hockin et al., 2012; Vanucci et al., 2012). The phase at which arrest occurs was shown to vary in different dinoflagellate species. For example, when completely N deprived, *A. cartarae* cells stop in G1, while *P. piscicida* cells stall at both G1 and G2/M phases (Olson and Chisholm, 1986; Lin et al., 2004). Of course, independent of the phase of cell cycle arrest, the resulting consequence is the same: cells cease dividing. At this point, organisms reach a state of stasis where the cells will either form cysts or store/remobilize their internal metabolites until N becomes available again. In photosynthetic eukaryotes, photosynthesis still occurs when N is limiting and C skeletons can accumulate. These were shown to be stored in the form of starch and/or as lipids in plants and green algae (Wingler et al., 2006; Wang et al., 2009). In dinoflagellates, accumulation of lipids in the form of triacylglycerol was found to be ~200% higher in N-limited conditions compared to control (Fuentes-Grunewald et al., 2012). In contrast, N-limitation in the HTD *Crypthecodinium cohnii* did not have any significant effects on the rates of lipid production (Pleissner and Eriksen, 2012). Thus, N stress may have different effects on the regulation of the enzymes involved in C storage between dinoflagellate groups.

Diatoms can also accumulate lipids, but only when cultured under very low N concentrations (Armbrust et al., 2004; Kroth et al., 2008; Hockin et al., 2012; Yang et al., 2013). At the early stages of N-stress, when the intracellular concentrations of ${NO}_{3}^{-}$, amino acids and proteins were about half the amount measured in N-replete cultures, no changes in C stores such as starch or lipids were detected in *T. pseudonana* (Hockin et al., 2012). It was proposed that diatoms had a greater tendency to remobilize rather than to store C compounds, because their OUC gives them the potential to increase the efficiency of N reassimilation from catabolic processes (Allen et al., 2006). An augmentation of reassimilation would lead to a greater use of C skeletons compared to organisms lacking a urea cycle (Hockin et al., 2012). This N remobilization hypothesis was suggested by the finding that recovery from N-stress in *P. tricornutum* was impaired in a RNA interference (RNAi) line having a reduced level of carbamoyl phosphate synthase, the enzyme catalyzing the first committed step of the OUC (Allen et al., 2011). Indeed, compared to wild type, this mutant accumulates less of most metabolites in the tricarboxylic acid (TCA) cycle and OUC. There are also reduced levels of Gln and other amino acids in the RNAi line, indicating that the OUC acts to link the TCA cycle and the GS-GOGAT pathway together. Thus, the OUC, being at the crossroad of catabolic and anabolic metabolism, was described as a distribution and repackaging hub for C and N compounds, which could have contributed to the success of diatoms in the modern oceans (Allen et al., 2011).

In some dinoflagellates, such as the MTD species *G. galatheanum*, *C. furca,* and *P. minimum*, N-limitation elicits a change in their N nutritional strategy, which switches from a primarily inorganic N uptake by transporters to uptake relying on both transporters and feeding mechanisms. A similar change in behavior was also recently shown to occur in free-living *Symbiodinium* spp. (Jeong et al., 2012), as while the cells ingested prey in both N-replete and N-limited conditions, the feeding rates were higher in the latter. In these experiments, ingestion of the raphidophyte *H. akashiwo* in ${NO}_{3}^{-}$ -limited conditions contributed up to 229% of *Symbiodinium* spp. daily body N compared to 105% in ${NO}_{3}^{-}$ -replete conditions. In other dinoflagellates, such as the type III MTD *P. piscicida,* when the amount of prey was reduced to ~1% of the predator population in the presence of high concentrations of DIN and DON (principally ${NO}_{3}^{-}$), uptake rates by transporters increased to levels similar to those cited for phytoplankton (Lewitus et al., 1999). Indeed, the highest rates of N uptake by transporters were comparable to the rates of N uptake by ingestion in *P. piscicida* cultures containing high amounts of prey (Lewitus et al., 1999). Clearly, some MTDs adapt to the N status of the environment by modifying their feeding behavior and altering the expression or activity of N transporters.

Transcriptional regulation of N metabolism is widespread in plants and algae (Galvan et al., 1996; Fernandez and Galvan, 2008; Lillo, 2008). In diatoms, comparative analysis of expressed sequence tags (ESTs) libraries under N stress in *T. pseudonana* and *P. tricornutum* revealed altered expression of numerous genes involved in N transport and assimilation (Maheswari et al., 2010). In this study, an ${NH}_{4}^{+}$ transporter was among the most up-regulated genes in N-depleted *P. tricornutum* cells. Similarly, in *C. fusiformis*, AMT1 and AMT2 transcript levels were highest in N-starved conditions (Hildebrand, 2005). Curiously, other transcripts, such as the one encoding NR in *C. fusiformis*, do not respond to variations in ${NO}_{3}^{-}$ concentrations, although its expression is inhibited by ${NH}_{4}^{+}$ (Poulsen and Kröger, 2005). These examples highlight the complex regulation of different N metabolism genes in diatoms.

Interestingly, in dinoflagellates, different high-throughput analysis of transcriptomes under N-stressed conditions revealed that transcriptional control also seems to occur in these organisms. Massively parallel signature sequencing (MPSS) in *Alexandrium* *fundyense* revealed that ~10% of the signatures were differentially expressed between N-stressed and P-stressed cells (Erdner and Anderson, 2006). This does not mean that 10% of the transcripts varied, however, as rapid amplification of cDNA ends (RACE) sequencing and mapping to known dinoflagellate genes showed that multiple signatures arose from sequence variants of individual genes. This redundancy was ascribed to the gene duplication events commonly described in dinoflagellate genomes (Erdner and Anderson, 2006). MPSS of *Alexandrium tamarense* cultures grown under various stresses was also performed, but in this study most of the differentially expressed genes were observed in comparisons between xenic and axenic cultures, rather than the N-limited ones (Moustafa et al., 2010). In *Karenia brevis*, a microarray comparison of N-limited and N-replete cells showed that ~11% of the probes differ between the two conditions (Morey et al., 2011). Interestingly, an ${NH}_{4}^{+}$ transporter, ${NO}_{3}^{-}$ /${NO}_{2}^{-}$ transporters and type III GSs were among the sequences up regulated under N-limitation. These experiments show that stress can induce transcriptional responses in dinoflagellates, which presumably act to fine tune dinoflagellate N metabolism in relation to environmental N status.

## CONCLUSION

Dinoflagellates use various strategies to acquire and assimilate N, depending on their trophic preferences and life styles. Some make an extensive use of transporters, others preferentially ingest prey and still others exploit both strategies equally. Enzymes for uptake and assimilation of N seem to share homology and kinetic properties with those reported in other photosynthetic eukaryotes. A remarkable feature both found in diatoms and dinoflagellates is their adaptability to changes in environmental N concentrations, particularly under stress conditions. Some mechanisms are common, such as symbiosis and transcriptional control; others are particular to dinoflagellates, like mixotrophy and vertical migration. With the advent of next-generation sequencing technologies, transcriptomic and even genomic tools will soon help in identifying and characterizing the molecular components involved in dinoflagellate N metabolism. Understanding how N is put in dinoflagellates will certainly help to better predict their behavior into our future anthropogenically altered aquatic ecosystems.

## Conflict of Interest Statement

The authors declare that the research was conducted in the absence of any commercial or financial relationships that could be construed as a potential conflict of interest.

## AUTHOR CONTRIBUTIONS

Steve Dagenais-Bellefeuille and David Morse wrote the text and figures were drafted by Steve Dagenais-Bellefeuille.
